# Remarks, Gleanings, and Extracts

**Published:** 1874-10

**Authors:** 


### Remarks, Gleanings and Extracts.

BY SWAN M. BURNETT, M.D., OF TENNESSEE.

*More Testimony on the Alcohol Question.*—Dr. H. C. Wood, in his late excellent "Treatise on Therapeutics," which proposes to consider the action of medicines from a physiological stand point, makes the following statements respecting the action of alcohol on the vital economy; and as he is an able experimenter, a close observer and a conscientious man, they are worthy of consideration at this time when so many and such diverse opinions are being promulgated, often by men who are in nowise worthy of confidence.

"From what has been said, it is certainly deducible that alcohol in small amounts is an *arterial* and *cerebral stimulant*, increasing functional activity in the nervous and circulatory apparatus; is a *food*, in the sense that it is destroyed in the system, and there performs a physiological office; is, when in sufficient quantity, a retarder of tissue changes, because it lowers heat independently of any action on the nervous system, and also probably checks excretion of carbonic acid and of nitrogen. If alcohol be oxydized in the body, and be a food, as it seems to me clearly proven, it must of course generate force, measurable by the modern standard of the heat-unit. A little calculation will show the importance, or rather the great amount, of the generated force. According to Dupré, one gramme of alcohol oxydized in the body evolves seven thousand one hundred and eighty-four units of heat, while the same weight of lean beef gives off only one thousand four hundred and eighty-four units of heat. It has been estimated that 9.3 ounces of lean beef equal to about two ounces of alcohol, will supply the necessary force to maintain the circulation and respiration of an average man for one day; that is, four ounces of strong spirit will suffice for this purpose. Since, to the ability of furnishing material whose consumption shall give power, is added the ability to restrict waste and to stimulate the functions of circulation and of the nervous system, it is evident that in alcohol we have a most important means of sustaining the system during the strain of an acute, exhausting disease."

Dr. Anstie, in the July number of the *London Practitioner*, also gives his "Final experiments on the elimination of alcohol from the body. From these researches we learn that in various ways all the excrementitious matter eliminated from the body was collected and examined with reference to the amount of alcohol that passed through the body unaltered. Such pains were taken in the carrying out of the experiments, and such is the honesty of Dr. Anstie, that we have now certain knowledge that all the alcohol taken into the system is not eliminated unchanged, but that a large part, a high per cent., is consumed in some manner in the body. He says:

"If, then, the subject of the elimination of unchanged alcohol may now, as I presume, be considered as closed, we can see our way to the discussion of other important questions respecting the physiological *rôle* of alcohol:"

"1. If alcohol be a force producing food, as by far seems the most likely, it is probably of great value in that capacity, on account of the rapidity with which its transformations take place. It is, however, abundantly certain that beyond a certain dosage it becomes a narcotic poison of a very dangerous character in every respect: not the least disadvantage being that it cannot be eliminated to any considerable extent."

"2. If alcohol does not disappear by oxidation, it must undergo some, as yet unknown, transformation, after which it must escape unrecognized in the excretions. I have heard various attempts to suggest such modes of disappearance, but nothing so far wears any air of probability."

"3. If alcohol be however indeed oxydized, and yet does not beget force which can be used in the organism, this would be the strangest possible discovery. Considering the very high theoretical force value of six hundred to eight hundred grains of absolute alcohol which millions of sober persons are taking daily, we may well be hopeless of any reasonable answer to the question: Why does not this large development of wholly useless force within the body produce some violent symptoms of disturbance?"

*A New Vaginal Speculum.*—Instruments for bringing into view the vaginal walls, and the neck and mouth of the uterus, are almost without number. But the very fact of their great number and variety proves that no one has yet been brought to such a degree of perfection as to answer all requirements. Hence almost monthly, either a new invention, or a modification of an old one, is brought to the notice of the profession through the various periodicals of the country. Some of these claim one thing as a peculiar point of excellence, and some another, and there are very few that combine more than two features of marked advantage over their competitors. An instrument, however, the invention of Dr. R. Knoffl, of this city, has recently been handed us for inspection and trial, which possesses so many traits of superiority, and is such a truly ingenious invention, that we think we would be doing our readers but justice in calling their attention to it. It is impossible, almost, to entertain a clear idea of the instrument without the aid of cuts or the instrument itself; but so far as a mere description will enable us, we will endeavor to convey to our readers an idea of the instrument and the manner of its operation.

Its essential parts are a double ring about two and a half inches in diameter, and a quarter of an inch broad, the two rings separated about one-eighth of an inch; and four fingers about four inches in length bent at a right angle at one inch and a half from one end—forming a figure like this:  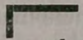 The long line of the rectangle is annealed towards the end, so that it can be bent to any required position; the rest is very stiff and firm. The fingers are nearly one inch broad, and fenestrated; their thickness a little less than eighteen inches, so as to slide easily between the two rings. The ring is the mouth of the speculum, and the fingers form the length of the instrument, when the short arm is inserted into the insterstice of the two rings. It will be readily seen that if four of these fingers are inserted around the circumference of the ring, that the walls of the vagina, and the os uteri—if the fingers are properly placed—will be brought clearly into view. But it has occurred, doubtless, to some one to ask, How are the fingers maintained in position? We havn't four hands, one for each of the instrument-fingers, and if we had, we would have to be possessed of another if we wished to manipulate at all. But one of the beauties of the instrument is its self-retaining power. In order to obtain this power, a well-known, but hitherto (so far as my knowledge goes), unused law of mechanics, is brought into play, and the elasticity of the vaginal walls, by pressure upon the end of the fingers, clamps the short arm tightly in the interstice, so that not only is the finger firmly fastened, but the whole instrument is retained *in situ*, without the intervention of any ex-traneous aid.

The advantages of this instrument are obvious upon a moment's reflection. In the first place, it is simple in construction, no screws or levers to get out of order. 2d. Is compact—can be carried in the vest-pocket. 3d. Is self-retaining. 4th. Gives a clear and unobstructed view of the vaginal walls, such as no instrument before the profession does, and is thus most admirably adapted for operations on these walls. 5th. Can be used in any position. 6th. Can be used in cases where the vagina is too small or irritable to permit the use of the large and cumbersome instruments generally employed. There are various accessories, as a tenaculum and its holder, etc.—which space forbids our describing—which makes this speculum, without doubt, the most complete of any yet brought out.

They are manufactured in this city, and any one wishing one can obtain it by writing to Dr. R. Knoffl, Knoxville, Tenn., where the instrument, with instructions for its use, will be sent. The price is $15.

*Traumatic Keratitis.*—Cohnheim, in his latest communication on the subject of inflammation, gives the following in regard to the inflammatory process in the cornea arising from trauma. It is to be remembered that the discovery which he has made respecting the phenomenon, or phenomena, we recognize as inflammation, is that the pus cell and the white blood corpuscle are identical; and that the latter, in order to become the former, has only to pass through the walls of the vessel and wander among the tissues. He and others have seen the white blood corpuscles actually passing through the walls of the vessels and thus wandering in the neighboring tissues.

The cornea is most admirably adopted for making observations respecting the wandering of these cells, and it is upon this tissue that many of his experiments have been made. His latest observations are recorded in the *London Medical Record* as follows:

"In every case in which, after an injury of the cornea, the tissue of the latter becomes infiltrated with more or less numerous pus-corpuscles, an alteration has preceded in the vessels of the conjunctiva next the cornea, consisting chiefly in the very marked injection of the vessels.

"That this injection of the conjunctival blood vessels is not the result of reflex-action can be proved, first, by the fact that traumatic keratitis can be very much favored by cutting the fifth nerve; and secondly, by the following experiment:

"In a rabbit, in which the facial nerve had been cut, in order to avoid the frequent movement of the eyelids, a portion of the cornea, two or three millimetres in diameter, is scraped off with a lancet-shaped knife; or the cornea is cauterized over a very small region with lunar caustic, care being taken that the caustic does not come in contact with the conjunctiva; or, finally, the cornea is cauterized with the knob of a red-hot probe. In all these experiments, generally no injection of the vessels of the cornea ensues, and the cornea remains perfectly transparent all round the injured part.

"In these cases, therefore, in which, after an injury to the cornea. an injection of the conjunctival vessels does ensue, and a subsequent opacity extends from the periphery towards the injured part of the cornea, there must be some other factor which produces the alteration of the vessels of the conjunctiva; it being proved by the experiments just mentioned that it cannot be due to reflex-action following irritation of the sensitive nerves of the cornea. Cohnheim takes it as very probable that in consequence of the traumatic injury, septic agents, produced by certain changes of tissue, probably chemical changes, gradually extend from the seat of injury towards the periphery until the vessels of the conjunctiva are reached, in the walls of which they produce an alteration, which gradually increases. The effects of this alteration are the above mentioned injection of the blood vessels and subsequent infiltration of the cornea with pus-corpuscles. With this assumption agrees very well the fact that often traumatic injuries which produce keratitis, the injection of the vessels of the conjunctiva, which is always the first symptom, does not immediately follow the injury, but takes always sometime for its development. The very immediate conjunctival hyperæmia in man, observable after traumatic injury of the cornea, is to be accounted for by the very vehement rubbing of the eye, and the increased and energetic movements of the eyelids. If, however, in a rabbit whose facial nerve has been cut through, keratitis be produced *e. g.* after inserting a fine silk thread in the centre of the cornea, there is nothing abnormal to be noticed in the first hour; in the third or fourth hour, conjunctival hyperæmia is observable, which gradually increases, its acme not being reached previous in the twenty-fourth or thirty-sixth hour."

In the same paper the author shows, by a series of experiments, that the cause of all these phenomena is an alteration in the walls of the vessels; and that in producing their effects, it is only necessary for noxious agents to come in contact with the walls of the vessels, contact with the other tissues not being necessary. The whole paper is interesting, as it sheds more light on the now most generally accepted theory of inflammation.

*Treatment of Hay Fever.*—Hemholtz, who has already made himself famous as a physiologist, physicist and mathematician, has recently gained new laurels by his researches, pathological and therapeutical, on the subject of hay fever, from which he is personally a sufferer.

He has described his case and his treatment of it in a letter to Prof. Binz, which is published in a recent number of *Nature.*

It appears that he has had attacks of hay fever, or asthma, every season for the last twenty years. It begins with great regularity during the haying season, and runs a definite course. Of course, a mind like his could not resist speculations as to the nature of the malady which caused him so much discomfort, to use the mildest term, and within the last five years his investigations have been in the direction of a microscopical examination of the mucus discharged from the nares.

He has found that the mucus discharged during an attack contains vibrios, which are not present in that discharged whilst he is free from the disease. They are very small, the length of the joints being only 0.004 m. m.

Seeing Binz's article on the poisonous action of quinia upon infusoria, he determined to try the influence of the remedy on his vibrios. Accordingly, he injected, by means of a pipette, a weak solution into the nares, and by movements of his head, while in a prone position, brought the solution in contact with all the points of the nares, posterior as well as anterior. The effect was all that could be desired. By a regular application at the beginning of the season, he was able to stop its development completely. He is now entirely free from the disease.

The proper way to make the application is by the "nasal douche," and the strength of the solution should be such as to occasion a slight degree of smarting. The solution should always be used tepid.

This plan of treatment, if successful, is to be preferred to Holmes' cure of "six feet of gravel," which is at present generally acknowledged to be the only certain one known to the profession.

*On the Value of High Powers in the Diagnosis of Blood Stains.* To question has more interest in its medico-legal relations than the determination of the source from which blood stains originate. It has been held by many, that after drying, the blood discs become so distorted that it is not possible to distinguish, with certainty, the blood of man from that of the lower animals—especially the ox, sheep, goat, etc.

Dr. J. G. Richardson, of Philadelphia, holds that it can be thus distinguished, and gives his reasons for so doing in the *American Jour. Med. Sciences* for July, 1874. He has contended for a long while, in the face of very high authority, that the blood-corpuscles are *cells*, and in this article he maintains that it is by virtue of being cells, that by proper treatment they can be made to approximate their normal size after drying.

"In conclusion," he says, "I submit, that the results of my experiments above narrated *prove that*, since the red blood globules of the pig, the ox, the red deer, the cat, the horse, the sheep, the goat, are all so much smaller than even the ordinary medium size of the human red disc, as measured in my investigations, *we are now able by the aid of the high powers of the microscope*, and under favorable circumstances, to positively distinguish stains produced by human blood from those caused by the blood of any of the animals just enumerated, and this even after the lapse of five years from the date of their primary production."

*Prolapse of the Vitreous Humor.*—In the *Le Mouvement Mèdical,* Piermé has recently contributed an important paper on the vitreous, based to a large extent upon experiments performed on lower animals; but containing besides, many important clinical and pathological data. His conclusions are as follows:

1. The vitreous humor is reformed readily and speedily, both in the eyes of man and the lower animals.

2. Prolapse of the vitreous humor is of greater danger when the wound is large, and naturally the gravity of the accident is greater in proportion to the amount of the loss.

3. The vitreous humor may become the seat of inflammation in consequence of a wound, and this inflammation may terminate by resolution, by suppuration, or may become chronic.

4. The immediate evil consequences of prolapse have generally their origin in the choroid, and the ultimate result is atrophy of the eyeballs.

*Iodoform in Venereal Ulcers.*—After cauterization with nit. acid, or actual cautery, as may be required, and the slough has come away, iodoform is used either in powder or suspended, thus: ℞. Pulvo. iodoform, gr. xlv.; glycerinæ, ℥iij; alcohol, ℥i. Applied once a day, every other day, or twicc a week, as may be required. *N. Y. Med. Rec.*

ACCORDING to some experiments of Vulpian, the small motion of the tongue, that remains after division of the hypoglossal, is due to the fibres of the *chorda tympani* that are distribueed to it. He looks upon the lingual as a purely sensory nerve.

THE largest blood corpuscle known belongs to the *amphiuma tridactylum* (congo cel). It measures 1-12 mm in its longest diameter. The red corpuscles of man measure 0.005 mm.

EPHRIAM CUTTER, in the *Boston Medical Journal of Chemistry,* recommend Indian meal as a vehicle of heat. He considers it superior to any of the poultices in use.

THE lungs of the Londoners are said to be much darker than those of any other people in the world—the supposed result of the fogs.

A SINGLE individual renders not less than 125 cubic feet of air unfit for respiration every twenty-four hours.

REMEDY for corns: Equal parts of glycerine and carbolic acid applied by means of a camel's-hair brush.

CARBOLIC acid is used in the treatment of lupus, and it is said with success.

*Doctors and the Poets.*—Euricius Cordus, over three centuries ago, sang thus:

> "Three faces wears the doctor: when first sought,
> An angel's—a God's the cure half wrought;
> But when the cure complete he seeks his fee,
> The devil looks less terrible than he."

Pope, with his universal knowledge of men and things, says in the same strain:

> "God and the doctor we alike adore,
> But only when in danger, not before;
> The danger o'er, both are alike requited:
> God is forgotten and the doctor slighted."

*Puerperal Amaurosis—Its Importance as a Symptom.*—Dr. H. W. Williams reports in the *Boston Med. and Surg. Jour.* two cases of his own and four from Weber, of St. Petersburg. He is of the opinion that it has some relation with puerperal convulsions, though the latter is not always preceded by the former. Albumen is not always found in the urine. Primipara are seldom attacked. He does not offer any special line of treatment; but advises that the physician watch closely for convulsions.

---

### REMARKS, GLEANINGS AND EXTRACTS

BY A. R. KILPATRICK, M.D., TEXAS.

*Vinegar Bitters.*—A number of years ago, an individual coming overland with a company to California, served as cook for the company, and was styled "doctor" on that account. He settled in Calaveras county, and labored as a miner, without much success. His attention was then turned to the medicinal qualities of the herbs growing about him, and he came to San Francisco with the idea of making and vending a preparation to be called "Indian Vegetable Bitters," and to contain no alcohol. He fell in with an enterprising druggist of this city, who saw money in the project and embarked in it. At the suggestion of the latter, the "Indian" was struck out, and as the new medicine got sour by fermentation, it was concluded to call it "Vinegar Bitters," and to identify it with the movement against alcoholic drinks. The mountain herbs were cast aside, and aloes, being a cheap bitter, was substituted. "Nine sick persons out of ten," said the druggist, "will be cured by purging; whereby the aloes." So the cook became a doctor, and the decoction became sour, and Dr. Walker's Vinegar Bitters began their career in the newspapers.—*Pacific Med. Jour.*

Such is a brief history of the California Vinegar Bitters, and something like this will be found to be the history of many other nostrums. We have known death to be caused by persons taking this stuff. In a recent article in the *Medical and Surgical Reporter* of Philadelphia, it is shown that the long list of these patent bitters and tonics have from seven per cent. to nearly fifty per cent. of alcohol in them, the Hostetter's Bitters standing about the top of the list.

*Hosteter's Bitters* took their origin thus: Hostetter is a Greek, who came to America a long time ago, and if he is alive yet he must be quite old. He served as a bar-tender some time at Natchez, Miss., and made a drink which was very palatable to his customers, and they would visit his bar and call for his bitters. Lawyers and merchants and planters from the adjacent country would call for them, and after a time some of the wealthy men got him to put up bitters for them by the gallon or demijohn, which they took home and used for a common drink. The bar-keepers on the famous steam packets had such frequent calls for Hostetter's Bitters, that they had to call on him to supply them with the drink in kegs full. These evidences of their popularity induced him to go fully and regularly into the business, and his name soon became notable. He had an establishment awhile in New Orleans, and soon found men with money to aid him, when he opened a large house in Pittsburg, Penn., where he still supplies his bitters in immense quantities. Of late years, I observe he styles himself DOCTOR in his advertisements, but I never have learned what medical college conferred the degree on him.

It is needless to say his bitters, for which he claims such great medical virtues, are made of ordinary cheap whisky, with a little bitter decoction and essence of orange-peel. He and his partner, Smith, if he is a real personage, have made quite a fortune out of the bitters, and could retire from the business and have enough to live on.

*Ergot as a Styptic.*—Since writing the article which appeared in the September number of the SOUTHERN MEDICAL RECORD, on "Ergot in Hæmorrhage," I have tried it to check the flow of blood from a deep, penetrating wound in the shoulder, made by the small blade of a pocket-knife. The wound was so small and so deep that it was not deemed advisable to attempt any ligation, but the flow, though not arterial, was constant and persistent for nearly a week after the infliction of the cut. The patient had been subject to frequent and exhaustive epistaxis, and had been relieved of that by the fluid extract of ergot. The place was very tender, and such that it was difficult to employ bandages for compression, so I determined to try the ergot; and as he was quite feverish, I combined quinine with it, and there was no more flow of blood after taking the first dose, still the medicines were continued longer for the tonic effect. I gave drachm doses of the tincture of ergot, as I had no fluid extract. This medicine is good also in diarrhœa, dysentery and hæmaturia.

It has been ascertained that ergot given to parturient women has no bad effect on the fœtus. Dr. Herschel reports a case of a child to which the nurse gave one drachm of the fluid extract by mistake, and which produced no bad effects.

*Orchitis.*—I have lately treated two cases of this disease which were caused by injuries, one being severely mashed by falling astride a log, the other caught on a wagon wheel, and subsequently made worse by lifting a heavy weight. There was febrile disturbance in both cases, requiring cooling laxatives. I used cold lotions of sugar of lead and belladonna in the first case without any benefit, and in the second with only very slight benefit. In the first case, one application of a strong solution of argenti nitras reduced the swelling and relieved the pain in a short time, and in the other the caustic was applied the second time after the lapse of three days, and acted as well as was desired. In a few days he was well. I am confident the nitrate of silver can be safely applied, and with prompt relief, to swelled testicle in cases of parotitis.

*Correction.*—*Xanthium Spinosum,* which I stated in the August number as the botanical name of the common *cockle bur,* is what is called *Tournefort,* and is not the native bur, but an exotic introduced into this country. *Xanthium Strumarium* is the real *bur, cockle bur, kuckold bur,* the pest of our fields and annoyance of our planters and laborers. Let me repeat, it is a good diuretic, a good hæmostatic, a good vesicant, and a certain cure for the bite of rattle snakes, given in decoction. Probably this correction is not really necessary, as very few people are particular about botanical names; but lest these few might be offended, or led astray by the mistake, I make the correction.

*Incontinence of Urine in Children.*—This troublesome affection, which has puzzled practitioners heretofore, is speedily relieved by the syrup of the iodide of iron, given in suitable doses at bed-time. A good combination is this: ℞. Syr. iodide of iron, ℥ivss.; chloral hydrate, ʒvss.; glycerin, ℥ss.; simple syrup, q. s. to make ℥viij. mixture. Dose, one teaspoonful at bed-time, and increased gradually to two spoonfuls. I have used the syrup of the iodide of iron alone, in from ten drops to a teaspoonful, with entire success, in cases which had resisted everything else, and other physicians say the same.

### REMARKS, GLEANINGS AND EXTRACTS

BY T. CURTIS SMITH, M.D., OF MIDDLEPORT, OHIO.

*The Value of Large Injections.*—The *London Medical Record* gives a summary of recent studies on injections. The following are the practical results:

1. Enemata, if sufficiently copious, will reach the small intestine, the ileo cæcal valve notwithstanding, provided there be sufficient propelling force, whether that be gained by a long column of fluid in the apparatus (as in the use of irrigators), or by the patient's position, with the pelvis elevated, favoring the descent of the fluid, or by repeated action of the injecting instrument. For ourselves, we never could see that the valvula Bauhini could ever be regarded as a real obstacle to injections from below, provided these were sufficiently copious; and we had made some experiments at different times, which had given ocular evidence of the extent to which injections had penetrated.

2. Hegar's and Mosler's experiments, and Wilbrand's case, show that it is neither necessary to use complex apparatus, nor to put the patients into awkward and perhaps dangerous positions; since from three to five feet of pipe, with a funnel at one end and a suitable nozzle at the other, is all the apparatus we need; and the patients simply lie upon the back, and the only pressure required is that of the column of fluid. We may here remark, parenthetically, that we fail to see the pertinency of the long discussion about atmospheric pressure, sub-atmospheric pressure, and the like; because, whether in or out of the body, making allowances for temperature and the varied characters of the gasses, we take it that atmospheric pressure may be regarded as a fairly constant unit; were it otherwise, learned professors and their poor patients would alike be crushed by the superincumbent air. The real pressure we have to overcome is that of the patient's muscles, aided in some cases by tense gasses in the bowel; for, if any one will insert a tube into the rectum before the injection has come away, he will see the fluid come out in jets or spirts when the patients strain, and less markedly so at every descent of the diaphragm. In certain positions of the body, there is also the difficulty of "making the water run up hill," when we are administering an enema in the ordinary manner, ingeniously overcome by Hegar and Mosler, either by making the water run down hill (or rather down a long tube) first, or by shifting the position of the patient.

3. The safety and efficiency, or the benign action, of large enemata of water, gruel, and the like, is strikingly shown by these experiments. We are strongly inclined, however, to believe that a very small quantity of soap, or of some neutral salt, is even less irritating to the mucous membrane than pure water alone. Except in rain water, or in water which has been frozen, man nowhere finds anything like a pure drink provided for him; and what is true in a superior sense, and, so to speak, *à priori*, is, we think, no less true *à posteriori*.

4. The caution impressed upon us by these experiments, not to allow air to get into the bowel, or as little as possible, is not an idle one. A very small quantity of air is not only intensely irritating to the bowel, but gives the greatest pain, almost agony in many cases. This fact was well known to our forefathers, although some of the so-called "trained nurses" of the present day, and many practitioners even, seem ignorant of it. Unless the precaution be taken to exclude air, the clyster is certainly not benign. To sum up all, large injections do reach the whole length of the large intestine, and beyond it; they are safe and speedy remedies for fecal accumulations, for some forms of intestinal obstruction (notably intussusception) and internal herniæ; for the treatment of intestinal ulcers, of hemorrhage from the bowels and diarrhœa; for worms, especially oxyurides and their congeners; as a means of stimulating and increasing the secretion of bile; and of introducing into the small intestine nutritious matters in a state easily susceptible of absorption.—*Med. and Surg. Reporter.*

*Reports on Bloodless Operations.*—The London *Medical Record* gives us the following brief abstract, made by Professor Esmarch, of his experience, during the past year of his experience in Kiel, of bloodless operations. The communication was made at the third sitting of the German Surgical Association, held in Berlin, April 8th to 11th, 1874.

From February 1, 1873, to April 1, 1874, above two hundred bloodless operations were performed in the Clinic at Kiel. Among them were the following:

*Amputations.*—Thigh, ten cases; one died with erysipelas and septicæmia.

Leg, eleven cases; one died with ditto.

Humerus, three cases; none died.

Total: twenty-four cases, with two deaths.

*Disarticulations.*—Shoulder-joint, one case; cured.

Hip-joint, one case; died of exhaustion.

Total: two cases, with one death.

*Resections.*—Hip-joint, three cases; one died of septicæmia.

Knee-joint, three cases; none died (one with subsequent amputation.)

Elbow-joint, two cases; none died (one with subsequent amputation.)

Total: eight cases, with one death.

Among the advantages of the bloodless method of operation, the following may be mentioned:

1. There is no loss of blood. (Anæmia produces a disposition to surgical accidents, by the tendency to coagulation, producing thrombosis, and leading often to pyæmia. Valsalva's method of treating aneurism acts by inducing greater coagulability of blood.)

2. It is a great advantage that sponges may be thus almost wholly avoided; for, with whatever care they are disinfected, one can never be absolutely certain that they are not sometimes the means of iufecting wounds.

3. The larger arteries and veins are less injured in the bloodless method than with ordinary digital or instrumental compression, the construction being uniform, circular, by means of the soft parts, and distributed over a large surface.

Esmarch knows of no dangers, nor of any bad results. Paralysis he has never seen to take place. It may be produced, however, by too forcible construction of the limb. Esmarch applies the apparatus himself, and does not trust his assistants, who are rather inclined to overdo their business. It is quite remarkable how comparatively small is the force necessary to arrest all flow of blood through the arteries. The first turns must not be made too tightly; every superimposed turn increases the compression very considerably.

Gangreene has not been observed in any instance by Esmarch. Some probable advantages of bloodless operations must be shortly hinted at.

1. Local anæsthesia may take place after a few minutes. Richet has, indeed, already recommended forcible constriction of the digit for the operation on ingrown nail. Richardson's ether-spray, or ice and salt, produces anæsthesia much more quickly in parts made bloodless, as the warmth of the blood is excluded. The temperature of the bloodless limb sinks immediately.

2. Diseased parts, bones, joints, can be very easily examined before performing an operation. Esmarch narrated very graphically how, on a recent occasion, he demonstrated a diseased joint before his class, pointing out the conditions which make an amputation uecessary.

3. Foreign bodies, splinters, needles, etc., are easily found.

4. Wounded arteries are easily exposed and secured. Antyllus' method is facilitated.

5. Operating without skilled assistants, or, indeed, without assistance at all, becomes possible in a way never before practicable.

Esmarch has received many letters of thanks from surgeons in various parts of the country.

6. It is suggested that death may be often averted, and cases of hemorrhage and transfusion avoided in anæmic persons, by bandaging the extremities, in order to temporarily drive the blood of one or two extremities into the body.—*Med. and Surg. Reporter.*

*New Researches on Diabetes.*—We learn that Dr. Pavy has obtained some experimental results which are likely to throw a new light on the subject of diabetes. He has found that the injection of fibrinated arterial blood in the portal system occasions a saccharine state of the urine. In one experiment, the urine after the operation contained 15 grains of sugar to the fluid ounce, and in others the quantity has amounted to nearly the same. In the counterpart experiment of injecting defibrinated venous blood into the portal system, the urine showed no signs of the presence of sugar. It thus appears that oxygenated blood passing to the liver causes an escape of sugar from the organ, and thence an accumulation in the system and discharge with the urine. It also appears that through the medium of the respiration of oxygen, he has succeeded in inducing a sufficiently oxygenated state of the blood to similarly give rise to the production of saccharine urine. He has further found that through the agency of the inhalation of puff-ball smoke an immediate and strongly diabetic state may be induced, and that the effect is accompanied with such a modification of the circulation that the blood flows through the vessels, as in the case after section of the sympathetic, without becoming properly de-arterialized. His experiments, he considers, suggest that, in diabetis of the human subject, the blood, in consequence of vaso-muscular paralysis, is allowed to reach the portal vein in an imperfectly de-arterialized condition, and thus determines the escape of sugar from the liver. We understand that his results are to be brought forward at the Royal Society as soon as its meetings commence.—*London Lancet. American Medical Weekly.*

*Digestion and Absorption in the Large Intestines.*—There has always been considerable uncertainty in regard to the question whether the large intestines are capable of digesting food. This power has been absolutely denied by several authors, as Blondlot, Frerichs, Braune, Funke, and Quincke, while others, as Zander, Schiff, and Eichhorst, have asserted quite as positively that the fluids of the large intestine are liable to digest both albumen and starch, the latter being first changed into sugar. Again, in regard to the form in which these substances may be absorbed in the bowels, opinions are also at variance; some claiming that the albumen must be first changed into peptone before it can be taken up, and others that fluid albumen may be as such absorbed directly. A similar diversity of opinion prevails regarding the ability of the intestines to absorb fats.

Some recent experiments by Drs. Czerny and Latschenberger, of Freiburg, throw some light upon these questions. The experiments were made upon a man who, through a malformation, had the anus situate in the left inguinal region, opposite sigmoid flexure. The rectum was so separated from the intestine above that matters could be introduced into it by the anus, and again washed out by a stream of water from above as from a retort. Various articles of food were thus introduced and experimented with, and the results which were obtained are thus stated:

" *The human large intestine and its secretions have no digestive action upon either coagulated or fluid albumen, nor upon fat.* Coagulated albumen, although left in the intestine for two and a half months, was not appreciably altered, nor was any change effected in fluid albumen in solution. After pouring in an emulsion of fat, the latter rapidly collected at the top, flowing together in large drops. Hence the large intestine not only has no emulsifying effect, but tends to destroy an emulsion already formed.

"The portion of intestine experimented upon absorbed 40 to 50 grammes of water within seven hours.

" *In the normal condition, fluid albumen in solution in water can be absorbed unchanged, as such, by the large intestine, and will be taken up in larger quantity the longer it remains.* Any state of irritation, as, for example, catarrh, hinders absorption or prevents it entirely. Chloride of sodium likewise interferes with absorption, but is taken up itself by the intestine, in spite of an irritated condition and impaired power of absorption. The albumen of the hen's egg is in an unfavorable form for absorption. When the white of an egg is introduced into the bowel unmingled with water only a very slight portion will be absorbed, and the same is true of it, even when beaten into a froth.

" Fat in emulsion is absorbed by the large intestine, and the amount actually taken up is proportional to the concentration of the emulsion and the length of time that it remains in contact with the absorbent surface.

"Starch, when hydrated, is absorbed by the large intestine, but whether directly as such or after being changed into sugar the experiment did not determine."

It was ascertained that 1½ grammes of albumen in a 4½ p. c. solution could be taken up in 24 hours. Since the portion of intestine experimented with was not over one-fourth the length the whole large intestine, it follows that the latter can absorb 6 grammes of soluble albumen in 24 hours. This quantity, however, is quiet inadequate to maintain the nutrition of the body, since 120 grammes is the quantity necessary for an ordinary person. It is presumed that by using more concentrated solutions of albumen the amount absorbed might be increased.— *Virchow's Archiv.—Allg. Med. Cent.-Ztg.—Kansas City Med. Jour.*

*The Automatic Man.*—Under this appellation is given, in the *Gazette Hebdomadaire*, of July 17, a curious case, which has come under the observation of Dr. Mesnet, of the St. Antoine Hospital. A young man, during the late war, had a portion of the left parietal bone, about eight centimeters extent, carried away by a ball. Hemiplegia of the right side was the result, but this gradually disappeared. For some time past he has been subject to attacks, lasting from twenty-four to forty-eight hours, attended by very extraordinary phenomena. During these, he seems to act like an automaton, walking continually, incessantly moving his jaw (*machonnant*), knitting his brow, and appearing absolutely insensible to all that surrounds him. Not uttering a word, he walks straight forward, and when he meets with an obstacle, stops short, explores it with his hand, and tries to pass on one side of it. Surrounded by a circle of persons, he stops at each and endeavors to pass by the intervals formed by their joined hands, then turns back, comes in contact with the next person, and resumes his round. All this time he never manifests the slightest consciousness, just as if he were in a state of somnambulism. He is absolutely insensible to pain, so that pins may be thrust through the cheeks or into the fingers, or very powerful electric shocks may be administered without the slightest sensibility being manifested. What, however, is very remarkable is, that by bringing him into relation with certain objects, we are enabled to determine in him the entire series of acts which are correlated with the sensation thus aroused. Thus, if a pen be placed in his hand, he seeks for ink and paper, and writes a letter in a good hand, in which he speaks very sensibly about matters that concern him. If a leaf of cigarette paper is placed in his hand, he feels in his pocket for the tobacco, rolls up the cigarette very adroitly, and, having found his match-box, lights it. If the match be extinguished just as it reaches the cigarette, he finds another, and that several times, till he is allowed to light his cigarette. If, at the moment when the match is extinguished another already lighted is presented to him in its place, it is impossible to induce him to light his cigarette by the substituted match. He allows his moustache to become burned without offering any resistance, but will not employ the light thus presented to him.

Among the various experiments devised by Dr. Mesnet, there is one which is particularly curious. The young man is a singer at concerts, by profession, and if gloves be placed in his hands, he immediately puts them on, and searches for paper. When a roll of this resembling music in form is given him, he places himself in the proper position and begins to sing. It would seem, in fact, that tactile sensation induced in him becomes the point of departure, and as if of escape of a series of acts correlated to their initial sensation—acts which he accomplishes automatically, without letting them deviate from their habitual and regular succession. Lastly, it is noted that, while in this singular condition, the patient steals all that comes within his grasp. If he touches any person, he feels for his watch-pocket, and invariably detaches the watch and puts it in his own pocket, from whence it may be removed without his making the slightest opposition. The crisis once over, he has no recollection whatever of what he has been doing, and becomes again perfectly reasonable.

The questions that such a case must give rise to for the reflection of the physician and physiologist, are striking. How, indeed, is such a fact to be characterized? And what idea is to be formed concerning the modifications of the functions of the nervous system which it exhibits? A no less interest must be felt by the medical legist, for evidently during these crises such an individual must be absolutely irresponsible. But how, under similar circumstances, are the facts to be ascertained?

What precedes is a mere sketch of some of the features of this curious case. Dr. Mesnet, armed with all the resources derived from a consummate experience in the study of mental diseases, has had it for some time under consideration, and will immedialely publish a memoir on the subject.—*Medical Times and Gazete*, July 25, 1874.—*Boston Medical and Surgical Journal.*

*Action of Nitrite of Amyl in Restoring Temporarily a Moribund Patient.*—I H., aged thirty-two years, laborer, entered the service of Dr. E. G. Janeway, at Bellevue Hospital, on May 27, 1874. The history obtained was to the effect that, during the summer of 1872 he first noticed his eyelids to be puffy, and his feet swollen. He continued to work, however, until five months before admission, when increasing debility, complicated with dyspnœa, palpitation, and loss of appetite, developed. When admitted, the patient was anæmic, very pale, and stated that his urine had been suppressed forty-eight hours. His feet and legs were very œdematous, and the rest of his body to a slighter extent. There was increased area of cardiac dullness, with a double murmur oves heart anteriorly, with the qualities of a friction sound of cardiac rhythm. There were also evidences of slight œdema of the lungs. The catheter removed only a few drops of urine from the bladder. Dry cups were applied over the lungs and kidneys, and half an ounce of infusion of digitalis, with half a drachm of acetate of potassa, given every four hours. The next morning the dyspnœa was improved, and about four ounces of highly albumninous urine passed. About noon, Dr. Janeway saw the patient for the first time. He was then unconscious. Pupils dilated. No response on touching the conjunctiva. Complete loss of pulse at the wrist, and breathing of a spasmodic character, such as occurs a few minutes before death. Some nitrite of amyl was obtained, and inhalations of five drops, cautiously increased to twenty-five drops, commenced. After the use of twenty drops, the pupils began to contract, and winking resulted from touching the conjunctiva. The pulse now began to be felt at the wrist. With the use of the last five drops he returned to consciousness, and the pulse at the wrist became full. He was now able to speak, and asked how long he had been faint, and whether or not he would pull through. The inhalations were now suspended, and half an ounce of brandy administered. A man was stationed at the bedside to notify the house-physician of the least approach of dangerous symptoms. In about fifteen minutes another attack occurred, and by the time the physician approached him he was dead.

There can be no doubt that in this case the amyl produced the temporary restoration from all but death, and the subsequent death in so short a time showed the gravity of the case.

The temporary restoration might be at times of great value, and, in a case where the lesions were not serious, might be prolonged into cure and permanent recovery.

Dr. Janeway has not read of any case in which such a near approach of death was warded off, and is of the opinion that, as a rule, it would not be judicious to give such large doses; but, from the fact that a certain amount remained on the towel, it is difficult to say how much in reality the patient inhaled. He does not believe that the nitrite would prove equally serviceable in cerebral hyperæmia producing unconsciousness, the case under observation being one of cerebral anæmia, with failure of the heart's action from pericarditis.

*Autopsy.*—Lungs œdamatous, with slight hydrothorax. Heart normal in size. Pericardium coated with fibrinous exudation of considerable amount. The sac contained a small quantity of serum. A microscopical examination of the tissue of the heart showed evidences of fatty degeneration in the portions beneath the pericardium. Liver, fatty. Kidneys of the large, white variety. Evidences of fatty degeneration, with increase of connective tissue. Cortical part of the kidneys anæmic. On one side there was a hydrocele.—*New York Med. Jour.*, July, 1874.—*Med News.*

*Influence of Chloroform in Labor upon the Fœtus.*—Dr. Zweifel, of the Obstetric Clinics in Strasbourg, has recently made some investigations that would seem to imply that the anæsthetic administered to women in labor has more effect upon the fœtus in utero than is perhaps generally admitted. Dubois has made the statement that anæsthesia of the mother causes increased rapidity of the fœtal heart-beats. The writer had often observed an appearance of icterus upon newly born children after the use of chloroform, but could not with certainty attribute it to the latter. His attention was at first seriously arrested by perceiving in the breath of an infant, born a few hours before, a distinct odor of chloroform. The child had been extracted while the mother was under the influence of the anæsthetic, but since the delivery had lain in a room by itself, where no chloroform had been used. Shortly after this, in order to determine positively whether the anæsthetic was conveyed to the fœtus through the maternal circulation, he instituted the following test: A fresh placenta that had just been expelled by a woman to whom chloroform had been administered for only about fifteen minutes, and more than an hour previously, was placed in a close-fitting vessel, having first been cleansed of all adhering clots. The following day, when the vessel was opened, a decided odor of chloroform was perceived, and further examination proved conclusively the presence of the drug. By still another test (the examination of the child's urine), the writer was able to establish the fact of the influence of the anæsthetic upon the fœtus. In conclusion, the writer observes that, since the use of narcotics in general are contraindicated in infants, it is an important question for obstetricians to decide, to just what degree anæsthesia may be carried in women in labor with impunity to the fœtus.—*Berliner Klinische Wochenscrift—New Yorh Medical Record.*

*Treatment of Chloroform Asphyxiation.*—Dr. Campbell, of Paris, recommends, in a late number of the *Journal de Therapeutique*, to place persons threatened with death from inhalations of chloroform head downwards and feet upwards for between ten and fifteen minutes. He considers that death arises from syncope due to cerebral congestion. The usual efforts at mechanical breathing, excitement of respiratory nerves, the drawing out of the tongue, insufflation into the lungs, etc., may be had recourse to concurrently. Dr. Campbell mentions only one case where this method succeeded. It was suggested by Nelaton during an operation performed at Paris by Marion Sims. It would appear that the late Professor Nelaton was the first surgeon who introduced this practice. The author also thinks that the inverted position tends to drive from the lungs and trachea pent-up vapors of chloroform, which were increasing the asphyxia.—*Dental Cosmos*, August, 1874.

*Atropia in Phthisical Sweating.*—Dr. James W. Williamson reports, in the *Lancet* of July 25th, the results of Dr. A. H. Hassall's experience with atropia in the night-sweating of phthisis. In sixteen cases in which the remedy was tried, he found the perspiration either wholly arrested or materially diminished; but in only one-fourth of the number was the effect permanent. The dose given was one-eightieth of a grain, in the form of pill, with extract of gentian. The quantity was increased, as it seemed necessary, to to one-sixieth or one-fiftieth of a grain. The watery solution of atropia was found liable to spoil, and inferior in its effects to the solid dose as administered. In some patients, the sensibility to the atropia rendered its use inadmissable; but in a large class of cases it is believed that it will answer a good purpose after other remedies have failed. The cases above mentioned were treated in the Royal National Hospital for Consumption, Ventnor.—*N. Y. Med. Jour.*

---

### REMARKS, GLEANINGS AND EXTRACTS

. BY LOCAL EDITORS.

*Treatment of Obstinate Constipation.*—Dr. Macario, of Nice, in a communication to the *Lyon Medicale*, observes that in treating constipation most practitioners confine themselves to enemata, laxatives, or more or less irritating purgatives, which in point of fact rather aggravate than cure the affection. He therefore wishes to make known what he says may be truly termed an "heroic" remedy, which he has employed during twelve years with such constant success that he cannot but regard it as infallible.

Constipation, as every one knows, may be produced either by intestinal excitement with deficiency of secretion (nervous constipation), or in consequence of deficient contraction of the muscular coat of the intestine. Here it is produced by atony or intestinal indolence, which bad anti-hygienic habits have induced and keep up. The prolonged contact of the fæces with the rectum blunts the sensibility of the mucous and muscular tissues, and the synergical contraction of the upper portions of the large intestine either does not take place or does so in an insufficient degree, constipation being the result. In *nervous* constipation the following pill should be given: Pure sulphate of iron ten centigrammes, socotrine aloes five centigrammes, atropine from one-third to one-half of a milligramme. In the *atonic* form, for atropine one centigramme of powder of nux vomica may be substituted. By the aid of these pills regular stools may be procured, even in the subjects of obstinate constipation due to *ramollissement* of the brain and chronic myelitis with paraplegia. Dr. Macario gives from one to three pills immediately after dinner, the object being to produce one easy, natural, non-diarrhœic evacuation. If more than this is effected, the dose is to be diminished, one or two pills sufficing in most cases. The use of these "antistyptic" pills ought not to be continued indefinitely, a longer interval being allowed to elapse between their administration in proportion as the constipation diminishes, it being of importance to allow the organs to resume their spontaneous action without any auxillary. If the constipation returns, the pills can be again had recourse to.

*Iced Clysters in Dysentery.*—Dr. Wenzel, having had occasion to treat a great number of cases of dysentery, has found the best remedy to consist in the injection of ice-water into the rectum. It is an inoffensive, economical treatment, and gives extremely satisfactory results. The first case the doctor treated in this manner was one of severe dysentery. There were intense fever, abdominal pains, excruciating tenesmus, and profuse sanguineous evacuations. To check the hemorrhage, injections of ice-water were ordered every two hours, which not only caused the sanguineous evacuations to cease, but also removed the tenesmus, enteric pains and fever. The beneficial effect of these injections was so evident that the patient urgently demanded their repetition whenever the pains threatened to reappear. Dr. Wenzel now considers this treatment more satisfactory than any other in acute cases, although in chronic cases it can only be expected to afford a temporary and palliative effect.—*L'Independente*, April, 1874—*New York Medical Journal.*

*Treatment of Varix by Injections of Hydrate of Chloral.*—In view of the fact that hydrate of chloral has the property of coagulating blood, Prof. Portà (*Revista de Med. e Chir.*) has instituted some experiments in the treatment of varicose veins, and arrived at the conclusion that the above remedy is the best for the relief of this condition. In proof of his assertion, he relates the histories of sixteen cases of varices of the legs, on which he has successfully operated. The quantity of chloral employed varies from one-third gramme to one gramme, and is not injected at once, but with intermissions; the coagulum forms immediately, and the patient must remain in bed for several days to guard against the occurrence of phlebitis; after this period the coagulum is gradually absorbed, and the veins atrophy, or, if remaining permeable, at least cease to be varicose. The accidents which are liable to occur under this plan of treatment are very rare, and not of a grave character: 1. Softening of the thrombus, usually transitory, and not retarding the final result. 2. Phlebitis, which is slight, and ceases in a few days. 3. Limited abscess. 4. A circumscribed eschar of gangrene of the skin, occurring in aged subjects. It is probable, observes the author, that this method of treating varices may be of use in regions of the body other than the limbs. He has already had one successful case of varicocele, and proposes to extend the treatment to subcutaneous nævi, arterio-venous aneurisms, and hemorrhoids.—*Revue de Thérap.*, 1874.

*Bismuth to Prevent Chaffing.*—Dr. Q. C. Smith (*Pacific Medical Journal*) says that a powder composed of oxide of zinc and starch, to prevent chaffing in infants, is no doubt a good remedy. But I beg to mention another, which I believe to be better, and one that long experience has taught me to value very highly. It is this: Take subnitrate of bismuth, in fine powder, and put in a thin flannel bag, and use as the old nurses use the old style "starch-bag," in dusting over the chafed parts, or those parts that are likely to or have already become sore, from any cause whatever. Should the parts be too tender for the use of the powder in this way, it should be sprinkled on plentifully. The good effect will, in many instances, manifest itself very quickly after the application. I have found bismuth an excellent external remedy for raw, blistered surfaces, and many other more or less superficial ulcerated surfaces.

*On the Use of Post-Partum Binders.*—At a recent meeting of the Obstetrical Society of Edinburgh, a somewnat remarkable paper was read by Dr. Cairns, opposing the use of binders after parturition, and, what is the strangest of all, his extraordinary views appear to have met with very general approbation from the members present.

The disadvantages in the use of binders enumerated by Dr. Cairns, are as follows:

1. That their application entails unnecessary trouble upon the *accoucheur*. Dr. Cairns confesses that when he first entered upon practice, it cost him more trouble to apply the binders, in many cases, than to deliver either the child or placenta.

2. That their application unnecessarily exposes the patient, which, if several persons are present, may thereby shock her moral sensibilities; it may, moreover expose her to currents of cold air, which, on her part, may lead to disastrous results.

3. Post-partum binders impede the circulation, slipping far above the region of the uterus, thus interfering with the venous circulation, and thus tending to aggravate two diseases very common in pregnant women, viz.: varicose veins and hemorrhoids.

4. They are rarely of proper form. They should properly extend from the ensiform cartilage to a considerable way beyond the nates.

5. In cases of post-partum hemorrhage, the patient may die before the binders can be removed in order to apply the proper remedies for its arrestment.

Dr. Cairns, in conclusion, compares parturition in civilized and uncivilized conditions, and those two with the parturition of the lower animals. The latter, he affirms, owing to their pendent bellies, evidently require binders much more than women.—*Boston Med. and Surg. Jour.*—*Clinic.*

*Treatment of Lumbago.*—Dr. G. V. Poore reports the case of a man, æt 35, who had the following history:

He had been accustomed to lathe-work, moving the treadle with his right foot, but had been unable to work for six years on account of a pain at first affecting the backs of the thighs and hips, but afterwards settling in the lower dorsal and lumbar regions. It was of a plunging, shooting character, much aggravated by the slightest movement, or even by the most gentle touch; he was bent almost double, and walked with the greatest difficulty. The intercostal muscles on both sides were affected, but there was no sign of any disease of the vertebræ or of the cord, and no particularly tender points were detected along the spinal column. He had been subjected to a great variety of treatments, but without any permanent relief having been afforded him.

The positive pole of a zinc-carbon battery was placed at the upper part of the spinal column in the median line, and the whole back, including the intercostal muscles, was sponged with the negative pole. During the application the patient was made to exercise his muscles rhythmically by bending, extending, and rotating the spine, and by frequent inspiration. The effects of this treatment were apparent from the first: the pain was greatly and immediately diminished, if not abolished, and in a short time all the extreme symptoms disappeared.

The important point was apparently the rhythmical exercise of the muscles, which improved their nutrition; but without the anodyne application of the galvanism such exercise would have been impossible.

*Galvanism in Post-Partum Hemorrhage.*—The following case may be considered of interest as furnishing an instance of the efficiency of a mode of treatment for the great opprobrium of obstetric art, which, if not new and little known, is at any rate far from being so widely employed as its numerous advantages would appear to indicate it should be.

The patient was in an extremely feeble state of health, and subject to epileptiform seizures. Convulsions came on during the first stage of labor, and could only be checked by keeping her under the influence of chloroform for some time. Failure of uterine action occurred before the os uteri was fully dilated; but, as it was sufficiently dilatable, forceps (Beattie's) were introduced, and delivery was accomplished. Still, the uterus did not contract, and, after the placenta was removed, hemorrhage could only be restrained by keeping the hand within the uterus. Grasping and kneading the uterus, cold effusion externally, and injections of cold water per vaginam, produced no effect. A dilute solution of perchloride of iron was freely injected into the uterus, but proved ineffectual. The employment of galvanism was then suggested as a *dernier ressort*, and one of Stohrer's portable coil machines was procured. An interrupted current of considerable intensity was directed through the uterus, one pole of the battery being applied to the abdominal walls immediately over the fundus, by means of a curved plate of copper, and the other placed in the cervix. Almost immediately firm contraction took place; and when the current was discontinued after a short time, the uterus remained securely contracted, and no further hemorrhage took place. The patient made a good recovery.—*British Medical Journal,* August 9.

*Sedative Power of the Bromide of potassium.*—A very striking example of this power has been published by Dr. Leriche, in *L'Union Méd.*, of July 19th, last. A man of thirty-five years had been suffering for some time from stricture of the spinchter ani. All kinds of applications were used, as opium, camphor, belladonna, baths, plugs, dilatation with the prepared sponge, etc., but to no purpose. The pain on evacuation was excruciating, and the finger could not be made to pass the contracted spincter. Dr. Leriche was then consulted and advised the cessation of all local applications. Rest and a light vegetable diet were prescribed, and thereupon the patient experienced considerable relief. As the latter expressed much fear at the proposal of an operation, thirty grains of bromide of potassium were given per diem. This dose was gradually increased to eighty. In a month all the disagreeable symptoms had disappeared, and the exercise of the functions of the rectum occasioned no pain whatsoever. It is plain that the case was one of spasmodic contraction. It would be worth while in strangulated hernia to try large doses of bromide of potassium.—*Lancet*.

*Lead in Subnitrate of Bismuth.*—A sample of subnitrate of bismuth from one of our most reliable manufacturing houses was recently found to be contaminated with nitrate of lead to such an extent that the impurity was detected at once by the sense of taste.

